# Hemosiderin quantification in hemophilic arthropathy using quantitative magnetic resonance imaging

**DOI:** 10.1038/s41598-025-99085-7

**Published:** 2025-04-24

**Authors:** Sam Sedaghat, Jame V. Luck, Annette von Drygalski, Eddie Fu, Jin Il Park, Kim Gina Gehling, Yajun Ma, Scott Ball, Eric Y. Chang, Jiang Du, Hyungseok Jang

**Affiliations:** 1 Department of Diagnostic and Interventional Radiology, University Hospital Heidelberg, Heidelberg, Germany; 2 Department of Orthopaedic Surgery, University of California, Los Angeles, Los Angeles, CA USA; 3 Department of Medicine, University of California, San Diego, San Diego, CA USA; 4 Department of Radiology, University of California, Davis, Sacramento, CA USA; 5 Department of Radiology, University of California, San Diego, San Diego, CA USA; 6 Department of Orthopaedic Surgery, University of California, San Diego, San Diego, CA USA; 7 Radiology Service, Veterans Affairs San Diego Healthcare System, San Diego, CA USA; 8 Department of Bioengineering, University of California, San Diego, San Diego, CA USA

**Keywords:** Hemophilia, Hemophilic arthropathy, MRI, Quantitative susceptibility mapping, Hemosiderin, Medical research, Translational research

## Abstract

The goal of this study is to quantify hemosiderin deposition in the knee joint tissues of hemophilic arthropathy (HA) patients using quantitative susceptibility mapping on MRI. Knee synovial tissues from HA patients and controls without hemophilia were included. The tissues underwent ultrashort echo time quantitative susceptibility mapping (UTE-QSM) and clinical MRI. HA tissues were processed histologically with Perl’s Prussian Blue (PPB) staining to identify iron contents. Seven regions of interest were drawn in each tissue, and the susceptibility values were tested. Moreover, the association between the estimated magnetic susceptibility and the iron contents quantified by histology was investigated. Nine synovial tissues were procured from total knee arthroplasty of hemophilia patients (males, 40.8 ± 9.0 years), and three synovial tissues were harvested from cadaveric knee joints of donors without hemophilia as controls (males, 72.0 ± 12.8 years). The estimated susceptibility values (ESVs) showed significant differences between HA and control samples. Accordingly, HA tissues presented a mean ESV of 0.48 ± 1.08 ppm and control tissues of -0.13 ± 0.12 ppm (*p* < 0.05). A significant linear correlation was found between the iron level quantified by histology (PPB stain) and the ESV estimated by UTE-QSM (*R* = 0.908, *p* < 0.01). There was a significant difference in the susceptibility in high load (HL) tissues compared to low load (LL) tissues (ESV = 5.57 ± 1.23 ppm for HL vs. 0.57 ± 0.85 ppm for LL, *p* < 0.001). Reliable hemosiderin quantification in joint tissues of HA patients can be achieved using MRI based on quantitative susceptibility mapping.

## Introduction

Hemophilia is an x-linked bleeding disorder characterized by deficiency of clotting factors VIII or IX^1^, afflicting approximately 1 out of every 30,000 male births^2^. One of the major complications of hemophilia is spontaneous bleeding. Joint bleeding typically begins in childhood and is often asymptomatic. A novel study revealed that almost one-third of the patients with mild HA develop joint bleeding^3^. Thereby, iron from red blood cells catalyzes hydroxyl radical production, which triggers a cascade that produces degradative enzymes and cytokines (e.g., IL-1 and TNF-α) by macrophages, leading to oxygen metabolite generation by chondrocytes and inhibition of proteoglycan (PG) synthesis. Damage in cartilage and other joint tissues may be directly induced by blood that subsequently causes an inflammatory response over and above that caused by hemosiderin^1,4,5^. HA can manifest in many different joints, with the ankle and the knee being the most involved joints in HA^1,6^. Identifying and quantifying the hemosiderin load of the knee joint early and reliably for more accurate diagnosis and treatment monitoring is of high clinical interest and importance. Also, early prophylaxis and prevention of later arthropathy is one key aspect of ongoing studies^7^. Accordingly, earlier diagnostics of hemosiderin depositions in the joints, especially in stages where prevention of later complications could be possible, would be highly beneficial for patients with HA.

MRI is a promising technique to quantify iron non-invasively because iron in biological tissues of the human body is predominantly paramagnetic^8^, which shortens T1 and T2 relaxation times. Iron concentrations can be estimated by measuring T1 and T2. Conventional R1 and R2 relaxometry, as well as more advanced quantitative susceptibility mapping (QSM) techniques, showed promising results in the assessment of iron contents^9–13^. Unfortunately, hemosiderin tends to accumulate in far higher concentrations in HA, which hampers accurate iron quantification due to its extremely short T2* decay. Moreover, a large portion of the signal from connective tissues such as tendon, ligament, and meniscus are invisible at the echo times (TEs) used in the techniques using conventional MRI sequences due to their short T2* decay, and hence those techniques are not able to detect nor quantify hemosiderin reliably.

One promising technique to acquire MR signals from the tissues with short T2 relaxation times is ultrashort echo time (UTE) MR imaging^14,15^. UTE imaging utilizes significantly shortened TEs, ~ 100x shorter than conventional spin-echo MRI, and it has been actively investigated in musculoskeletal disorders such as osteoporosis and osteoarthritis^16^. Due to its capability to directly resolve the short T2 signal, UTE-based relaxometry has been used to characterize joint tissues. For example, T2* relaxometry can be used to characterize collagen fiber networks in short T2 tissues^17^, and T1ρ relaxometry can characterize proteoglycan contents in musculoskeletal tissues^18^. Moreover, UTE-based QSM (UTE-QSM) has been investigated to assess bone mineral density^19^.

Recently, the feasibility of UTE-QSM in assessing hemosiderin in HA has been demonstrated^20^. In the UTE-QSM technique, multiple images at significantly shortened TEs are acquired to resolve signals from the connective tissues and hemosiderin-laden tissues. The feasibility study showed that UTE-QSM with fat-water signal modeling allows quantitative assessment of iron concentrations in the knee and ankle joints. In this study, we use the recently proposed UTE-QSM MRI technique to quantify and assess hemosiderin depositions in joint tissues of patients with hemophilic arthropathy, as imaging biomarkers for that purpose do not exist yet.

## Methods

### Specimen

All experiments were performed in accordance with relevant guidelines and regulations. All experimental protocols were approved by the Institutional Review Board of the University of California, San Diego. Informed consent was obtained from all subjects and/or their legal guardian(s). For this ex vivo study, hemosiderin-laden synovial tissues were collected from total knee arthroplasty of hemophilia patients with a history of hemophilic arthropathy. Additionally, synovial tissues were harvested from cadaveric human knee joints without a record of hemophilia. The tissues underwent a single freeze-thaw cycle after the harvest and were then prepared in individual 30 mL tubes with saline for the subsequent MRI. After the MRI, the tissues were fixed in formalin for 72 h and underwent histological processes.

### MRI

MRI was performed in a 3T clinical scanner (MR750, GE Healthcare, Milwaukee, WI, USA) using a three-inch surface coil (GE Healthcare). The MRI protocol consisted of a UTE-QSM based on UTE sequence with 3D cones trajectory, as well as clinical sequences including T1-weighted fast spin echo (T1w-FSE), T2-weighted fast spin echo (T2w-FSE), and T2*-weighted gradient recalled echo (T2*w-FSE) sequences.

**Fig. 1:**
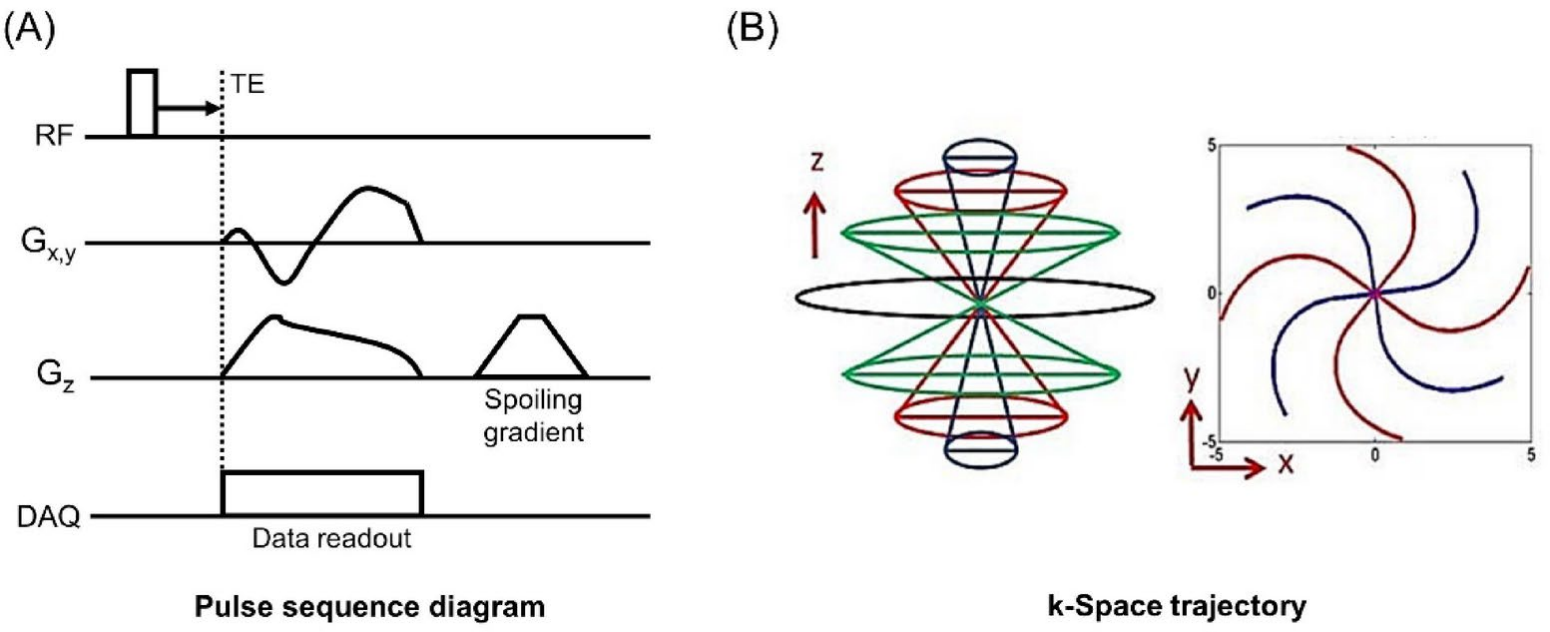
UTE imaging for QSM. (**A**) MRI pulse sequence diagram and (**B**) k-space trajectory.

Figure 1 shows the UTE sequence and k-space trajectory used for the UTE-QSM. To acquire UTE and GRE images at variable TEs with short echo spacings, the imaging was repeated with the readout gradient with different time delays, which yielded 13 echoes. Table 1 shows the MR imaging parameters.

**Table 1:** MRI parameters.

UTE-QSM	T1w-FSE
• Flip angle (FA) = 13° • TR = 17 ms • TE = 0, 0.1, 0.2, 0.4, 0.6, 0.8, 1.2, 1.7, 2.2, 2.7, 3.2, 3.7, and 4.4 ms • Field-of-view (FOV) = 60 × 60 × 16 mm^3^ • Matrix = 200 × 200 × 32 • Readout bandwidth (rBW) = 125 kHz • Scan time = 279 s per scan	• FA = 111° • TR = 742 ms • TE = 8.3 ms • FOV = 60 × 60 • Matrix = 200 × 200 • Slice thickness = 0.6 mm • Number of slices = 10 • rBW = 83.3 kHz • Echo train length (ETL) = 3 • Number of excitation (NEX) = 2 • Scan time = 205 s

T2w-FSE	T2*w-GRE
• FA = 111° • TR = 2786 ms • TE = 70 ms • FOV = 60 × 60 • Matrix = 352 × 256 • Slice thickness = 0.6 mm • Number of slices = 10 • rBW = 83.3 kHz • Echo train length (ETL) = 18 • NEX = 2 • Scan time = 173 s	• FA = 15° • TR = 200 ms • TE = 9.8 ms • FOV = 60 × 60 • Matrix = 300 × 260 • Slice thickness = 0.6 mm • Number of slices = 10 • rBW = 50 kHz • NEX = 4 • Scan time = 211 s

### Ultrashort echo time quantitative susceptibility mapping

The acquired images were processed using the morphology-enabled dipole inversion (MEDI) QSM pipeline^21^. A water-fat signal model based on six fat peaks was incorporated to deal with the fat signal, which is a major confounding factor for musculoskeletal QSM^20,22^. The resultant total field map was processed using a projection onto dipole fields (PDF) algorithm to yield a local field map^23^. For the MEDI algorithm, a regularization parameter of 500 was used.

### Histology

Each synovial tissue was placed in a 1.1’’ x 1.60’’ x 0.27’’ histology cassette. All the histology cassettes were fixed in 10% neutral-buffered formalin for 48 h. All the cassettes were processed overnight for 16 h in the Sakura Tissue-Tek VIP processor. After the processing, the tissues were embedded in paraffin and subsequently sectioned at 5 μm on the slides and ready for iron staining. All the slides followed the standard Perls’ Prussian Blue (PPB) staining protocol, in which the slides were immersed in the potassium ferrocyanide and hydrochloride acid mixture solution for 20 min and counterstained in Nuclear Fast Red solution for 5 min. If the iron (i.e., hemosiderin) is present in the tissue, it would appear blue. All the stained slides were scanned using the Zeiss Axioscan 7 automated slide scanner using the 40X Brightfield Imaging modes.

### Statistics and data analysis

A radiologist and an MRI physicist with over ten years of experience analyzed the resultant susceptibility map and the corresponding histological images. First, the MR images and the resulting susceptibility maps, as well as histology images, were inspected to assess imaging artifacts, image quality, and visual similarity between MRI and histology. Subsequently, regions of interest (ROI) analysis was performed on the estimated susceptibility maps from UTE-QSM and histological images. The seven ROIs were manually drawn per tissue using a susceptibility map or histology image using Matlab and ImageJ. Between two groups of tissues (normal vs. hemophilia), the mean susceptibility values were compared using the Mann-Whitney-U-Test. With the tissues from four HA patients, which underwent both MRI and histology, Pearson’s linear correlation between the mean susceptibility values from UTE-QSM and iron density (intensity of blue stain) from histology was tested. A p-value under 0.05 was statistically significant for all tests. Statistical analysis was done using the IBM-SPSS version 28.0 software package (IBM, Armonk, NY, USA).

## Results

### Baseline data

In this study, nine HA tissues (all males, 40.8 ± 9.0 years) were procured from the Department of Orthopaedic Surgery at the University of California, Los Angeles, and three control tissues (all males, 72.0 ± 12.8 years) were harvested from three cadaveric knee joints. Table 2 summarizes the information on this study’s human knee joint synovial tissues. All tissues underwent MRI, and four HA tissues underwent histological process.

**Table 2:** Demographic information for the tissues included in the study.

	Control (*n* = 3)	Hemophilia (*n* = 9)
Sex	3 males	9 males
Age	72.0 ± 12.8 year-old	40.8 ± 9.0 year-old
Type of hemophilia	N/A	“A” for all tissues
Degree of hemophilia	N/A	“Severe” for all tissues

### MR images

**Fig. 2:**
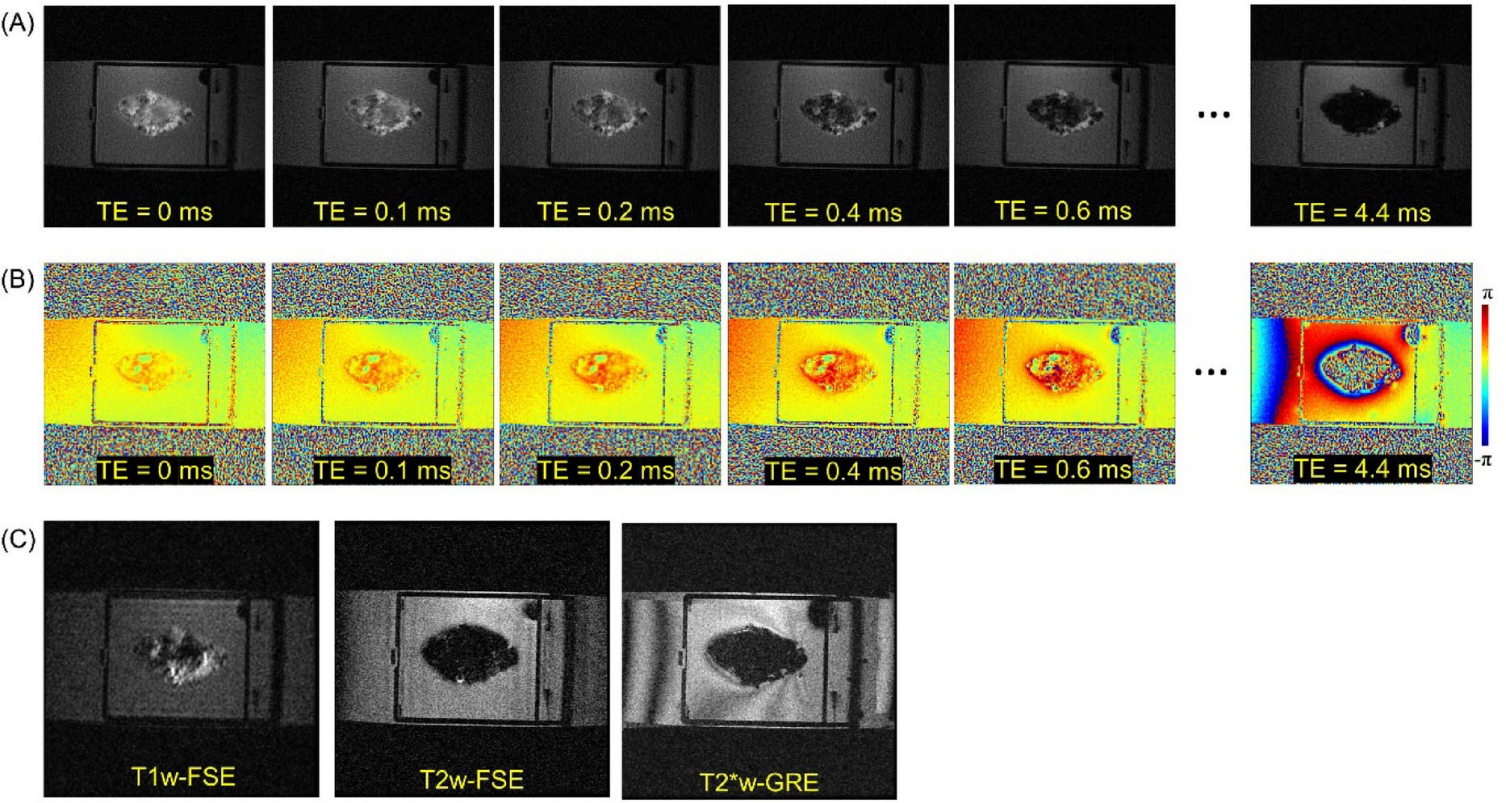
UTE-QSM and the corresponding clinical MRI images. (**A**) The magnitude and (**B**) the phase of UTE-Cones images at different TEs, and (**C**) the corresponding clinical T1w-FSE, T2w-FSE, and T2*w-GRE images.

Figure 2 shows input UTE-QSM images at different TEs and the corresponding clinical MR image. Due to the highly accumulated hemosiderin in the synovial tissue, a strong blooming artifact with signal nulling is exhibited in the clinical MRI images (Fig. 2C), which obscures the morphological assessment of hemosiderin deposition. In contrast, UTE images show the dramatically reduced blooming artifact (Fig. 2A). Figure 2B shows the acquired phase images with different TEs, which show strong phase variation in the regions where hemosiderin is deposited due to the strong magnetic susceptibility.

### Controls vs. HA tissues

**Fig. 3:**
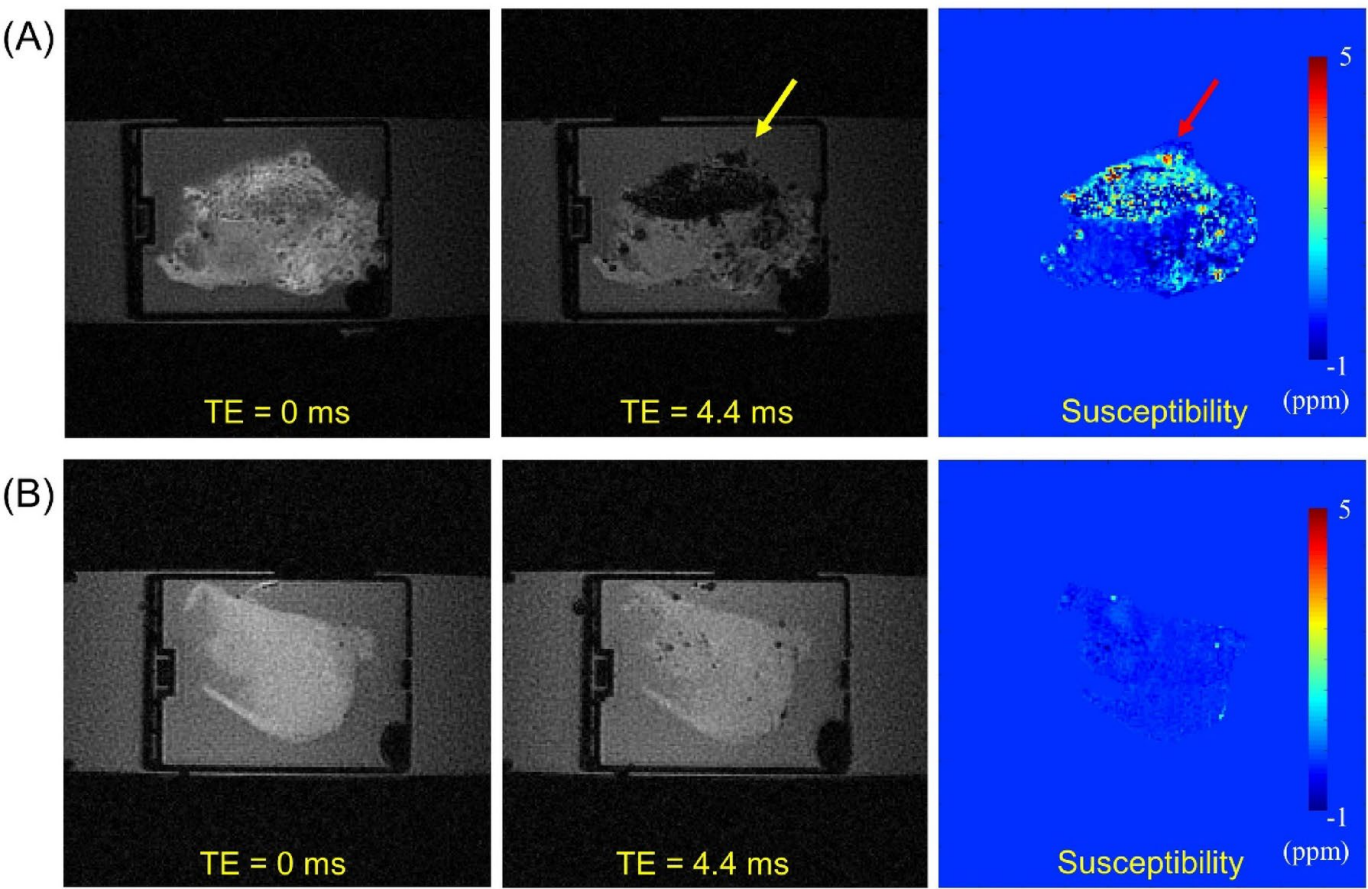
Comparison between HA (**A**) and control (**B**) tissues. The HA tissue exhibits strong signal decay between two TEs (0 and 4.4 ms) (a yellow arrow), which is also clearly detected in the susceptibility map (a red arrow). The control tissue does not show such strong signal decay nor estimate of high susceptibility.

Figure 3 shows the representative susceptibility maps from the control and HA groups. The synovial tissues from the HA group showed dramatically increased susceptibility values due to the accumulated hemosiderin. In the ROI analysis, the mean estimated susceptibility value (ESV) from the control group (21 ROIs) was − 0.13 ± 0.12 ppm, while the ESV from the HA group (63 ROIs) was 0.48 ± 1.08 ppm. Accordingly, the two groups showed a significant difference in the susceptibility values (*p* < 0.05).

### MRI vs. histology

In visual inspection, the 3D susceptibility maps from UTE-QSM were highly similar to the corresponding histological findings for all tissues.

**Fig. 4:**
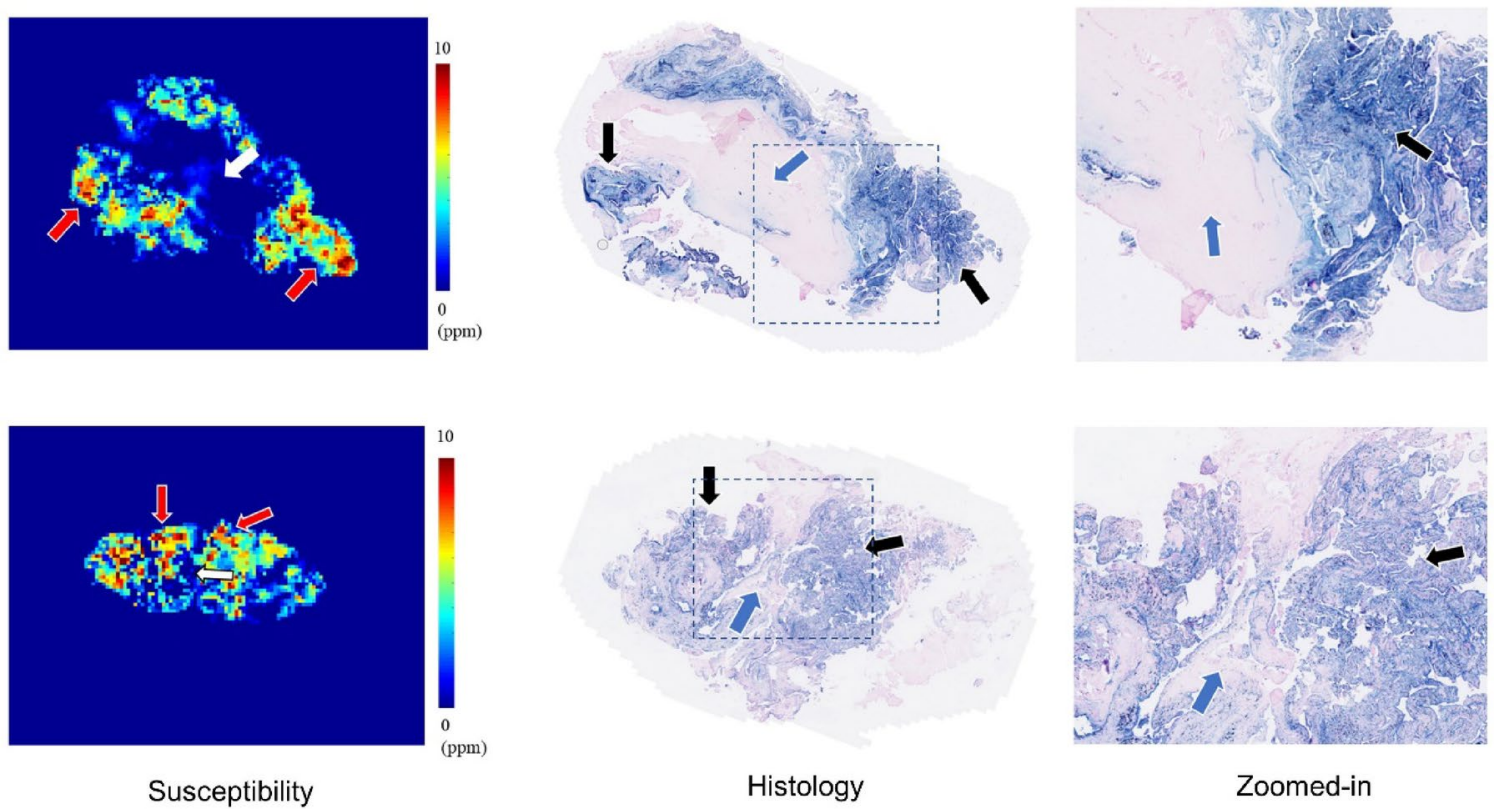
UTE-QSM and histology from representative hemophilia tissues. The elevated susceptibility values (red arrows) in the UTE-QSM correspond well with the strong blue signal in the histology (black arrows). The regions with low susceptibility values (white arrows) can be identified in histology (blue arrows).

**Fig. 5:**
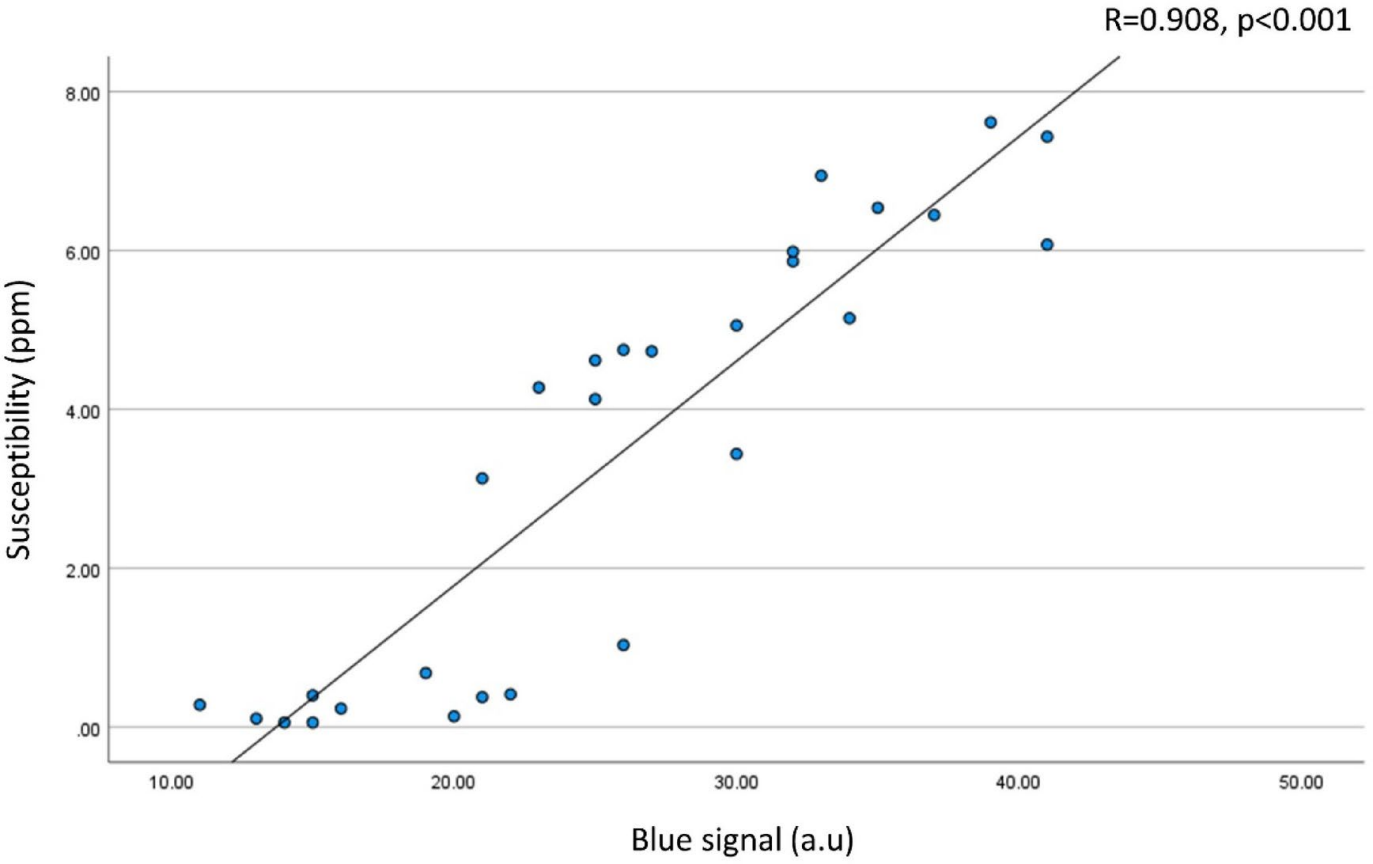
Susceptibility vs. blue signal. A strong positive correlation was found between the susceptibility from UTE-QSM and the blue signal from PPB-stained histology (*R* = 0.908 with p-value < 0.001).

Out of 28 ROIs from four HA tissues that underwent both MRI and histology, 16 ROIs showed high iron load, while 12 ROIs showed low iron load on UTE-QSM and histology. The mean ESV from UTE-QSM was 5.57 ppm (SD: 1.23 ppm) for high iron load and 0.57 ppm (SD: 0.85 ppm) for low iron load tissues. We found a significant difference in the ESV in high iron load tissues compared to low iron load tissues (*p* < 0.001). Figure 4 shows representative examples of HA tissues in UTE-QSM with the corresponding PPB-stained images. Figure 5 shows the scatter plot between susceptibility values and the corresponding PPB intensity in all ROIs. Overall, the ESVs from UTE-QSM showed a strong positive correlation with respect to the PPB signal from histology, with a Pearson’s correlation of *R* = 0.908 (*p* < 0.001). The cutoff for differentiating between high iron load and low iron load was seen at a susceptibility of 3.28 ppm.

## Discussion

In this study, we assessed and quantified hemosiderin depositions in joint tissues of patients with hemophilic arthropathy on quantitative MRI using a UTE-QSM approach.

Hemophilia is a genetic bleeding disorder caused by a missing or defective clotting factor and is classified as a rare disease^5^. Patients with hemophilia face the risk of uncontrolled bleeding, which can be fatal. However, the most common clinical manifestation is the development of hemarthrosis, particularly in the knees, ankles, and elbows^5^. The risk of hemarthrosis is notably higher in individuals with severe hemophilia, where even minimal trauma can trigger bleeding^24^. Repeated joint bleeding eventually leads to synovial hypertrophy and hemosiderin deposits, further stimulating synovial hypertrophy and swelling^25^. The recurrence of hemarthrosis ultimately results in a degenerative and irreversible condition known as hemophilic arthropathy^5,26^. As the degeneration in HA and, accordingly, the hemosiderin deposition increase, more disability occurs. Therefore, it is critical to establish imaging techniques to assess hemosiderin deposition in the human joints and quantitatively evaluate their concentration, eventually determining disease severity. However, no established imaging method exists that can reliably measure hemosiderin concentrations and depositions within the synovial tissue. The idea behind our concept is that earlier diagnostics of hemosiderin depositions in the joints can have a tremendous outcome for HA patients. Detecting early HA changes could help delay or even prevent later complications, usually accompanied by total knee replacement. Trying to achieve early diagnostics of HA changes within the joints is, therefore, a quite novel and innovative approach.

MRI already plays an important role in evaluating various soft tissue conditions and changes, ranging from brain tissue changes^27,28^ to tissue changes due to HA^10,20,29^. Nevertheless, highly accumulated hemosiderin is invisible on conventional MRI due to the strong susceptibility (known as blooming artifacts) and the short T2 decay^20,30^. Also, QSM, which has demonstrated its capability to quantify iron deposition in various body parts, such as the brain^31–34^, liver^35^, and kidney^36^, is not directly applicable to HA due to the highly accumulated iron, which causes a ~ 10-100x larger magnetic susceptibility. For example, in brain diseases, iron deposition leads to less than a 100 ppb increase in Alzheimer’s disease^37^, while over one ppm increase is commonly observed in HA^20^, resulting in dramatically shortened T2* decay. UTE-QSM can enhance sensitivity to iron deposition at high concentrations, making it effective in quantifying hemosiderin in HA, both in low and high concentrations, as demonstrated in this study. In addition, UTE-QSM can help assess tissues with strong susceptibilities, such as bone^19^. T2* relaxation time has been explored as a quantitative marker for assessing hemosiderin accumulation, with its reciprocal, R2*, showing a promising linear correlation with iron content^10^. However, while R2* is sensitive to iron, it also reflects signals from both paramagnetic and diamagnetic tissues, making it less specific to hemosiderin.

This is where UTE-QSM comes into play. Unlike R2*, UTE-QSM offers a more targeted approach, reliably distinguishing hemosiderin with exceptional contrast. This advantage arises because most tissues in the human body are diamagnetic, allowing UTE-QSM to highlight hemosiderin deposits with much greater specificity. This specificity makes UTE-QSM a powerful tool for pinpointing hemosiderin, providing more precise insights into conditions where iron deposition plays a critical role.

To quantify iron deposition using UTE-QSM, it is essential to calibrate the relationship between actual iron concentrations and magnetic susceptibility. In the literature, phantom experiments with iron-oxide nanoparticles have demonstrated a high degree of linearity between these two parameters^11,30,38^. Using the established reference equation, measured susceptibility can be converted into actual iron concentrations. One significant challenge is the partial volume effect, which can lead to underestimation of iron levels due to the cancellation between diamagnetic and paramagnetic susceptibility sources. For instance, calcium in human bone has a strong diamagnetic property (~ -1 ppm). When hemosiderin and bony tissue occupy the same voxel, their susceptibility sources can cancel each other, resulting in underestimation of the measured values. This effect hinders the accurate quantification of hemosiderin. A potential solution involves utilizing prior knowledge of tissue susceptibility values to detect elevated levels relative to expected tissue characteristics. However, this method is not truly quantitative due to variability in baseline susceptibility values within the complex structures of joint tissues. A more promising approach involves a novel QSM technique called susceptibility source separation, which has been validated in the brain for separately detecting myelin and iron^39–41^. In future studies, we aim to explore this technique to achieve truly quantitative iron assessment in HA.

While imaging modalities such as ultrasound can reliably detect the presence or absence of intracavitary hemarthrosis^42^, they lack the ability to visualize hemosiderin accumulation or its clearance within tissues, such as hypertrophied synovium^43^. This is a critical limitation, as iron deposition is recognized as a key driver of inflammation and joint destruction in hemophilia. Clinical data suggest that up to 30% of HA cases involve asymptomatic bleeding that remains undetected and untreated^44^. Furthermore, studies in hemophilic mouse models have demonstrated impaired synovial iron clearance following induced joint bleeding. Thus, hemosiderin accumulation or delayed clearance may serve as a marker of subclinical joint bleeding and/or dysfunctional iron metabolism. From a clinical perspective, the ability to quantitatively assess hemosiderin is therefore essential for evaluating the efficacy of prophylactic therapies. In this context, tools enabling longitudinal quantification of intra-articular hemosiderin are crucial for optimizing treatment strategies and preventing the progression of hemophilic arthropathy. Our study demonstrated that UTE-QSM MRI reliably detects hemosiderin deposits and offers a quantification accuracy that closely parallels traditional histological methods. This advancement opens the door for non-invasive hemosiderin quantification, eliminating the need for surgical specimen collection. Furthermore, the ability to detect and assess early hemosiderin deposition before any visible hypertrophic changes occur represents a significant step forward in disease staging and monitoring. This early detection capability could facilitate timely therapeutic interventions, potentially improving patient outcomes by addressing pathological changes at a stage where they are more manageable. Early hemosiderin detection could delay the disease’s progression and help shift the therapeutical focus to non-surgical treatments^45^.

The proposed UTE-QSM technique can provide a snapshot of hemosiderin deposition in the joint, which can aid in treatment planning, such as prophylaxis with clotting factor replacement^46^. Since joint bleeds can be asymptomatic and go unnoticed, monitoring hemosiderin levels and distribution after joint bleeds is critical. This applies to both acute and chronic bleeds. We expect the regular monitoring with UTE-QSM MRI can offer a better understanding of the outcomes of prophylaxis. However, the clinical utility of hemosiderin quantification remains largely theoretical and is currently confined to pre-clinical research. While hemosiderin is one of several features detectable on UTE-QSM MRI, its independent role in predicting the progression of arthropathy has not yet been established. This underscores the need for further investigation in larger, longitudinal studies, which we have planned for future research.

In our future studies, the current ex vivo protocol will be further optimized to achieve the clinically feasible scan time, including the reduced number of TEs, interleaved multi-echo acquisition, and other acceleration techniques such as compressed sensing and deep learning^47,48^. We will apply it to a cohort of hemophilia patients and healthy controls.

There are several limitations of this study. Although we only demonstrated the results with tissues harvested from the knee joint of HA patients, UTE-QSM would be feasible for any affected joints, such as the elbow and ankle. Moreover, only ex vivo tissues were included in this study since histology can only be performed after total knee arthroplasty. Another possible approach is to compare in vivo MRI pre-arthroplasty and histology post-arthroplasty. However, the limited spatial resolution caused by limited scan time in the in vivo MRI may not be the optimal choice for this study, comparing UTE-QSM and histology. Furthermore, it may not be easy to co-register the in vivo MR images with ex vivo histology images. Another limitation is that the study used synovial tissues from cadaveric knee joints as a control. Tissues harvested from total knee arthroplasty would serve as a better control group.

In conclusion, we showed that hemosiderin deposition in joint tissues of hemophilia patients can be reliably detected and quantified using a novel quantitative MRI method called ultrashort echo time quantitative susceptibility mapping. This method could act as a biomarker for evaluating the stage of hemosiderin deposition in the tissue.

## Data Availability

The datasets used and/or analysed during the current study available from the corresponding author on reasonable request.
